# Updates on Antibody Drug Conjugates and Bispecific T-Cell Engagers in SCLC

**DOI:** 10.3390/antib15010004

**Published:** 2026-01-04

**Authors:** Kinsley Wang, Kyle Taing, Robert Hsu

**Affiliations:** 1 College of Medicine, University of Arizona College of Medicine—Phoenix, Phoenix, AZ 85004, USA; kinsleywang@arizona.edu; 2 Department of Internal Medicine, Huntington Health, Pasadena, CA 91105, USA; kyle.taing2@huntingtonhealth.org; 3 Division of Medical Oncology, Department of Medicine, University of Southern California Norris Comprehensive Cancer Center, Los Angeles, CA 90033, USA

**Keywords:** small cell lung cancer, antibody drug conjugate, T-cell engager, DLL3, tarlatamab

## Abstract

**Background/Objectives**: Small-cell lung cancer (SCLC) is an aggressive neuroendocrine malignancy characterized by rapid proliferation, early metastasis, and near-universal relapse after initial therapy. While chemo-immunotherapy modestly improves first-line outcomes, survival after progression remains poor and highlights the urgent need for biomarker-directed strategies. **Methods**: A comprehensive literature search was conducted using major medical databases looking at key relevant studies on SCLC antibody studies. All authors reviewed the literature, assessed study quality, and interpreted the results from each study. **Results**: Recent advances in antibody–drug conjugates (ADCs) and T-cell engagers (TCEs) have transformed therapeutic development by targeting antigens selectively expressed on SCLC cells, enabling more precise and potentially durable tumor control. DLL3 has emerged as the most clinically relevant target to date, with the bispecific TCE tarlatamab demonstrating meaningful and durable response, manageable cytokine-release toxicity, and ultimately achieving accelerated FDA approval for previously treated extensive-stage SCLC. Concurrently, DLL3-directed ADCs have shown variable efficacy, underscoring the importance of payload selection, linker chemistry, and antigen density. Beyond DLL3, next-generation ADCs targeting TROP2, B7-H3, and SEZ6 have reported encouraging early-phase activity, including response rates exceeding those of existing second-line cytotoxic options, though myelosuppression, interstitial lung disease, and hepatic toxicity remain key considerations. **Conclusions**: Collectively, these emerging immunotherapies illustrate a shift toward antigen-specific targeting in a disease historically defined by limited therapeutic innovation. Continued optimization of antigen selection, payload and linker engineering, and biomarker-driven trial design will be critical for translating early promise into durable clinical benefit and reshaping the treatment landscape for SCLC.

## 1. Introduction

Small-cell lung cancer (SCLC) is an aggressive neuroendocrine tumor that accounts for roughly 13–15% of lung cancers worldwide, with an estimated 250,000 new cases and more than 200,000 related deaths each year [1]. Its incidence closely follows historical smoking patterns with a several-decade lag, declining in many high-income regions but rising in low- and middle-income settings. Despite geographic variation, SCLC remains uniformly aggressive, marked by rapid proliferation, early vascular and lymphatic dissemination, and a tendency for two thirds of patients to present with metastatic disease at diagnosis. Although initial responses to chemotherapy can be striking, relapse is almost universal and long-term survival remains poor, with five-year survival rates below 7% [2,3,4]. Unlike non-small-cell lung cancer (NSCLC), where oncogenic drivers have transformed treatment, SCLC lacks clear targetable alterations, and limited tissue availability has historically constrained biologic study, reinforcing the need for new therapeutic strategies [5].

Although SCLC is still treated as a single disease, molecular studies show that it is biologically heterogeneous. Although transcription-factor defined subtypes (ASCL1, NEUROD1, POU2F3, and the inflamed SCLC-I group) have been described and show distinct therapeutic sensitivities, these profiles remain investigational and are not used in clinical practice [6]. Subtype-specific responses to immunotherapy and targeted agents have been suggested, but current evidence is retrospective and unvalidated. Similarly, biomarkers such as SLFN11 have shown associations with improved survival and sensitivity to DNA-damaging therapies, yet they have not been prospectively validated for treatment selection [7].

Limited-stage SCLC (LS-SCLC) can be treated effectively with aggressive multimodality therapy that combines platinum-etoposide chemotherapy with concurrent thoracic radiotherapy, achieving initial response rates of 70–90% [8,9]. Select early-stage, node-negative patients may undergo surgical resection or stereotactic body radiotherapy followed by adjuvant chemotherapy, while consolidation durvalumab for two years after chemoradiation has recently been shown to prolong both overall and progression-free survival [10,11]. Because brain metastases develop in around 60% of LS-SCLC patients within three years of diagnosis, either close MRI brain monitoring or prophylactic cranial irradiation remain standard for stage IIB-IIIC patients with good response to initial therapy, with neurocognitive toxicity as a key consideration [12,13,14].

For extensive-stage SCLC (ES-SCLC), first-line therapy remains platinum-etoposide combined with a PD-L1 inhibitor such as atezolizumab or durvalumab, which improves overall survival by roughly two to three months compared with chemotherapy alone [2,3]. Most patients progress within several months despite ongoing maintenance immunotherapy, and studies adding further checkpoint inhibitors, PARP inhibition, or DLL3-directed ADCs have not shown a survival benefit [15,16]. Recently, the IMforte trial reported that combining lurbinectedin with atezolizumab during maintenance led to longer progression-free and overall survival than atezolizumab alone, albeit with higher toxicity, leading to FDA approval in October 2025 as a maintenance option for ES-SCLC [17,18]. Prophylactic cranial irradiation is controversial, with uncertain survival benefit compared to MRI surveillance [14]. Once relapse occurs, prognosis is poor, and most patients derive only modest benefit from currently available second-line cytotoxic therapies, making enrollment in clinical trials the preferred strategy whenever possible [19]. These limitations underscore the need for biologically targeted approaches, highlighted by the recent accelerated approval of tarlatamab for post-platinum, post-immunotherapy disease and the growing interest in DLL3-, TROP2-, and B7-H3-directed therapies as more rational strategies for relapsed ES-SCLC.

Despite advances in first-line chemoimmunotherapy, about 90% of patients with ES-SCLC ultimately experience relapse, and outcomes after progression remain poor. Resistance to immune checkpoint blockade in SCLC is multifactorial and has been attributed to low tumor mutational antigen presentation, an immunosuppressive tumor microenvironment, and rapid lineage plasticity following treatment exposure [20,21,22]. In addition, antigen escape through loss or downregulation of target expression, as well as on-target off-tumor effects related to shared antigen expression on normal tissues, represent important challenges for antigen-directed immunotherapies in SCLC [23]. In LS- SCLC, integration of immunotherapy with chemoradiation has recently demonstrated meaningful clinical benefit, as shown in the ADRIATIC trial, where consolidation durvalumab following concurrent chemoradiation significantly improved survival compared with placebo [11]. Together, these observations highlight both the context-dependent efficacy of immunotherapy in SCLC and the need for novel immune-based strategies capable of overcoming intrinsic and acquired resistance in advanced disease.

**Figure 1:**
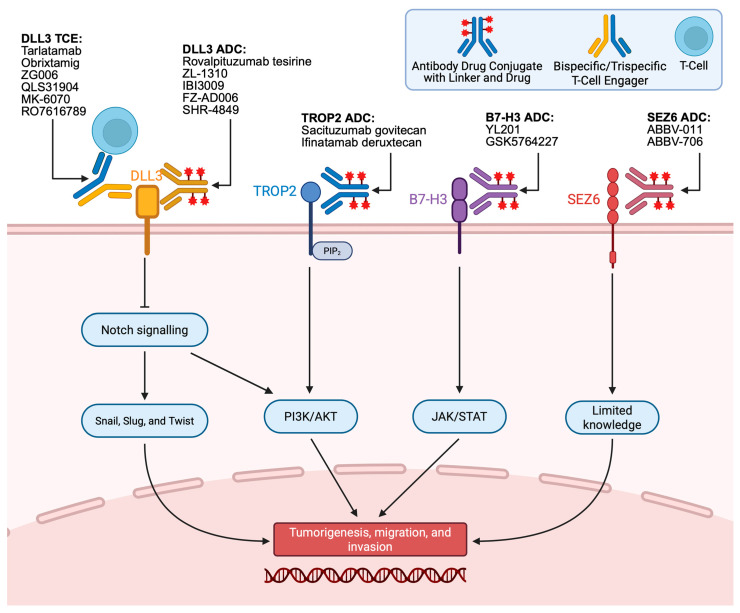
Various Targets and Therapeutics in Small-Cell Lung Cancer.

Standard second-line options offer only modest and short-lived benefit, underscoring the lack of effective salvage therapy. These limitations have accelerated the development of next-generation targeted therapeutics, including bispecific T-cell engagers and antibody–drug conjugates directed at DLL3, B7-H3, TROP2, and other emerging antigens. Relevant therapeutics and their clinical results are shown in Table 1, and their corresponding targets are illustrated in Figure 1. Although no validated biomarkers exist to match these therapies to individual patients, the increasing number of agents against novel molecular targets represents an important direction for future treatment of extensive-stage and relapsed SCLC.

## 2. DLL3 T-Cell Engagers

Delta-like ligand 3 (DLL3) is an atypical member of the Notch ligand family that has emerged as a promising therapeutic target in SCLC [24,25,26,27]. Normally absent in adult tissues, DLL3 is aberrantly expressed on the surface of approximately 80% of SCLC cells, where it functions as a cis-inhibitor of the Notch signaling pathway, preventing receptor activation and downstream transcription of genes such as HES1 and HEY1 [28,29]. As shown in Figure 1, this inhibition maintains the undifferentiated, highly proliferative neuroendocrine phenotype characteristic of SCLC and is transcriptionally driven by ASCL1, a key regulator of neuroendocrine differentiation [30]. Beyond impaired differentiation, DLL3 overexpression promotes tumor growth, epithelial-to-mesenchymal transition, and metastasis through the regulation of Snail, Slug, and Twist, and may interact with pathways such as Wnt/β-catenin and PI3K/Akt to enhance tumor survival [31,32,33]. Its minimal expression in normal tissue and consistent presence on SCLC cells provide a favorable therapeutic window, leading to the development of DLL3-directed therapies.

T-cell engager (TCE) molecules have emerged as a promising immunotherapeutic approach for DLL3-positive SCLC. These agents link CD3 on cytotoxic T cells to DLL3 on tumor cells, triggering targeted immune activation and tumor lysis independent of antigen presentation. DLL3-directed TCEs differ in molecular architecture, with bispecific constructs containing DLL3- and CD3-binding domains, whereas trispecific agents incorporate an additional functional binding domain that may further modulate T-cell activation (Supplementary Figure S1).

Tarlatamab (AMG 757) is a bispecific TCE that binds to both DLL3 and CD3, leading to T-cell-mediated tumor lysis [34,35]. In preclinical models, it has demonstrated potent and selective cytotoxicity against DLL3-expressing SCLC cells, including those with very low antigen density. The first-in-human trial (Phase 1, DeLLphi-300, NCT03319940) established the safety, pharmacokinetics, and preliminary efficacy of Tarlatamab in heavily pretreated SCLC [35]. Treatment produced an objective response rate (ORR) of 23.4%, with a median progression-free survival (mPFS) of 3.7 months and median overall survival (mOS) of 13.2 months, while maintaining a manageable safety profile dominated by low-grade cytokine release syndrome (52%) and infrequent grade ≥ 3 events, supporting further clinical development. Building on these findings, in the phase 2 DeLLphi-301 trial (NCT05060016), patients with previously treated SCLC received tarlatamab at 10 mg or 100 mg intravenously every two weeks [36]. ORR was 40% and 32% in the 10-mg and 100-mg cohorts, respectively, with durable responses lasting ≥6 months in 59% of responders and median progression-free and overall survival of 4.9 and 3.9 months. Cytokine-release syndrome was the most common adverse event (50–60%). Based on DeLLphi-301, tarlatamab gained accelerated approval by the Federal Drug Administration (FDA) for use in ES-SCLC with disease progression or after platinum-based chemotherapy [37]. DeLLphi-304 is a randomized phase 3 trial testing tarlatamab versus standard of care (SOC, including topotecan, lurbinectedin, or amrubicin) second-line chemotherapy in patients with relapsed SCLC (NCT05740566) [38]. In interim analyses, tarlatamab demonstrated superior activity to SOC, with an ORR of 35% versus 20%, mOS of 13.6 months versus 8.3 months, and mPFS of 4.2 months versus 3.7 months. On the basis of these findings, the FDA granted full approval for tarlatamab in the relapsed setting [39].

Two other phase 3 trials are currently underway. DeLLphi-306 is an ongoing randomized, double-blind, placebo-controlled phase 3 trial evaluating consolidation tarlatamab after chemoradiation in limited-stage SCLC, aiming to determine whether DLL3-directed T-cell engagement can improve progression-free and overall survival in this population (NCT06117774) [40]. DeLLphi-305 is an ongoing randomized phase 3 trial evaluating whether adding tarlatamab to durvalumab maintenance after first-line platinum plus durvalumab can improve survival outcomes in extensive-stage SCLC (NCT06211036) [41].

Additional trials aim to investigate tarlatamab as a first-line therapy. DeLLphi-303 (NCT05361395) is evaluating tarlatamab as a first-line maintenance strategy following initial platinum-based chemotherapy [42]. Meanwhile, DeLLphi-312 (NCT07005128) is evaluating tarlatamab in the first-line setting by comparing tarlatamab plus SOC (including durvalumab, carboplatin, etoposide) versus SOC alone, with the potential to establish tarlatamab as part of initial therapy for extensive-stage or limited-stage SCLC.

Obrixtamig (BI 764532) is another bispecific DLL3/CD3 IgG-like TCE under clinical investigation. In the phase 1 study (n = 168, NCT04429087), it was evaluated in heavily pretreated DLL3-positive SCLC, extrapulmonary NECs, or LCNEC, showing a manageable safety profile dominated by mostly low-grade, early, and reversible cytokine release syndrome. Across all regimens, ORR was 23% with a median DOR of 8.5 months. Step-up dosing achieved higher activity, including ORR of 21% in SCLC, supporting continued development in DLL3-expressing neuroendocrine tumors [43].

ZG006, a trispecific DLL3 × CD3 construct, is also in a phase 1 trial (NCT05978284). Preliminary results show substantial antitumor activity in relapsed SCLC (n = 23), with ORR 60.9% and DCR 78.3% of evaluable patients treated at 10–60 mg [44]. Responses were maintained across DLL3 expression levels; low or medium DLL3 expression achieved ORR of 66.7%. Toxicity was dominated by early, transient CRS and was manageable with no treatment-related discontinuations.

Other DLL3-directed TCEs include QLS31904, a DLL3 × CD3 bispecific antibody in phase 1 testing (NCT05461287). MK-6070 and RO7616789 are other trispecific DLL3-targeted agents currently being evaluated in phase 1/2 (NCT06780137) and phase 1 (NCT05619744) studies, respectively.

## 3. DLL3 Antibody–Drug Conjugates

Rovalpituzumab tesirine (Rova-T) is a DLL3-targeted ADC consisting of a DLL3-specific IgG1 monoclonal antibody linked via a protease-cleavable linker to a pyrrolobenzodiazepine (PBD) dimer cytotoxic payload, which is released following lysosomal cleavage following DLL3-mediated internalization (schematic of structure and mechanism of action shown in Supplemental Figure S1). The first-in-human phase 1 study (NCT01901653) studied Rova-T in recurrent high-grade SCLC (n = 74) as second-line therapy [45]. Dose escalation established a maximum tolerated dose of 0.4 mg/kg every 3 weeks and a recommended phase 2 dose of 0.3 mg/kg every 6 weeks, with ORR of 18% and higher activity (~38% ORR) in DLL3-high tumors but was limited by thrombocytopenia, liver enzyme elevations, pleural effusions, and photosensitivity. In the subsequent TRINITY phase 2 trial in heavily pretreated (third-line or later) SCLC (NCT02674568), activity was modest, with ORR 12–14% and a mOS of 5.6 months [46]. Similar adverse events, including cytopenias, serosal effusions, and skin reactions, were common. The phase 3 TAHOE study (NCT03061812) tested Rova-T as second-line therapy in DLL3-high advanced SCLC but showed inferior survival compared with topotecan (mOS 6.3 vs. 8.6 months), leading to early termination and discontinuation of further clinical development [47].

ZL-1310 is another DLL3-targeted ADC that links a DLL3-specific monoclonal antibody to a topoisomerase I inhibitor payload via a cleavable linker designed to undergo lysosomal cleavage following DLL3-mediated internalization, enabling intracellular payload release. It is being evaluated in a two-part phase 1 study (NCT06179069) for relapsed/refractory ES-SCLC [48]. The dose-escalation cohort (n = 28) showed an ORR of 68% and 93% disease control, with activity observed across all dose levels, including in patients with brain metastases, low DLL3 expression, and one patient previously treated with tarlatamab. The safety profile was manageable, with cytopenias and rare interstitial lung disease as the main grade ≥ 3 toxicities, supporting continued evaluation to refine dosing and confirm clinical benefit.

IBI3009 (NCT06613009), FZ-AD005 (NCT06424665), and SHR-4849 (NCT06443489) are other DLL3-targeted ADCs currently being evaluated in phase 1 clinical trials, all employing topoisomerase I inhibitor payloads delivered through cleavable linkers that undergo lysosomal cleavage following DLL3-mediated internalization. IBI3009 uses a DLL3-specific monoclonal antibody, whereas FZ-AD005 incorporates an Fc-silenced anti-DLL3 antibody conjugated to a DXd (topoisomerase I inhibitor) payload via a protease-cleavable valine–alanine dipeptide linker to avoid off-target toxicity [49,50]. SHR-4849 employs a humanized anti-DLL3 IgG1 antibody linked to a topoisomerase I inhibitor payload through a cleavable linker [51].

## 4. TROP2 Antibody–Drug Conjugates

Trophoblast cell surface antigen-2 (TROP2) is a type I transmembrane glycoprotein involved in cellular adhesion and proliferation as well as in calcium signaling [52]. Various other names for this protein include tumor-associated calcium signal transducer 2 (TACSTD2) and epithelial glycoprotein-1 (EGP-1). TROP2 dysregulation has been implicated across multiple malignancies, including triple-negative breast cancer, urothelial carcinoma, endometrial cancer, and lung cancers, with IHC studies demonstrating moderate to high TROP2 expression in SCLC. In SCLC specifically, TROP2 is frequently expressed on the epithelial surface, including on tumors with variable neuroendocrine differentiation [53,54]. Its consistent surface localization and efficient antibody-mediated internalization has supported the development of TROP2-directed ADCs, most notably sacituzumab govitecan, which is FDA approved for metastatic triple-negative breast cancer per results of the ASCENT study in cases with disease progression after two lines of chemotherapy showing both a median PFS and OS benefit, hormone receptor-positive/HER2-negative breast cancer per results of the TROPicS-02 study in previously treated cases that had disease progression with a CDK4/6 inhibitor, endocrine therapy, and a taxane with at least 2 lines of prior therapy, and previously treated advanced urothelial carcinoma per the TROPHY study in patients with disease progression after chemotherapy and PD-1/PD-L1 inhibitor, where high and consistent TROP2 surface expression and efficient ADC internalization have translated into clinically meaningful response rates [55,56,57]. As such, TROP2 has emerged as a viable ADC target, with multiple platforms that have been developed and tested thus far in treating SCLC.

Two different ADC platforms have emerged with an objective of targeting the TROP2 glycoprotein: Sacituzumab govitecan (SG) and the deruxtecan (DXd) family. SG consists of a humanized anti-TROP antibody conjugated to SN-38 (the active metabolite of a topoisomerase I inhibitor known as irinotecan) [58]. Alternatively, the DXd family, which includes ifinatamab deruxtecan (I-DXd), consists of an anti-TROP antibody conjugated to deruxtecan, a derivative of another topoisomerase I inhibitor known as exatecan [59]. Both SG and I-DXd essentially operate by delivering a potent topoisomerase I inhibitor as its payload directly to tumor cells.

Preclinical trials, including in vitro and xenograft models, have demonstrated that TROP2 ADCs induced apoptosis and tumor regression in TROP2-expressing SCLC cells [60]. The TROPiCS-03 study, however, has recently emerged as a key phase II trial, in which ES-SCLC patients previously on immunotherapy and platinum therapy (n = 43) were treated with SG (10 mg/kg on days 1 and 8 of a 21-day cycle) (NCT03964727). Efficacy was evaluated in both patients with platinum-resistant and platinum-responsive disease. Through this study, SG ultimately demonstrated promising efficacy as a second-line therapy for ES-SCLC irrespective of platinum sensitivity, with an ORR of 41.9% and mDOR of 4.7 months [58]. The IDeate-Lung01 trial also recently emerged as a phase II trial in which patients with ES-SCLC (n = 137) who previously failed previous lines of therapy (median of 2 lines) were assigned to receive 12 mg/kg I-DXd (NCT05280470). This trial yielded a confirmed ORR of 48.2%, mDOR of 5.3 months, median time to response of 1.4 months, mPFS of 4.9 months, and 9-month overall survival estimate of 59.1% [59].

The above studies also demonstrate that toxicities associated with the TROP2 ADC mirrors the known toxicities of its payload. Namely, myelosuppression (including neutropenia) as well as diarrhea, the classic toxicities of the topoisomerase I inhibitor family, predominate as the key side effects for both TROP2 ADC platforms. Furthermore, polymorphisms in the UGTIA1 enzyme may increase SN-38 exposure, which may subsequently increase toxicity risk in specific patients receiving the SG formulation [61]. Alternatively, ILD and pneumonitis remain the most clinically significant risk in those receiving the I-DXd ADC. These toxicities may be appropriately addressed with frequent CBC monitoring, early G-CSF support for neutropenia, and discussion of diarrhea management as well as ILD symptoms.

## 5. B7-H3 Antibody–Drug Conjugates

B7-H3 (also known as CD276) is a type 1 transmembrane glycoprotein that acts as a co-inhibitory molecule in the tumor microenvironment (TME) by impairing T-cell activation. Namely, B7-H3 deregulation has recently been found to activate multiple pathways, including JAK/STAT, mTOR, MAPK, and NF-κB, leading to increased tumor aggressiveness [62]. Furthermore, a higher B7-H3 expression has been shown to clinically correlate with worse survival outcomes in many tumor types, including lung [63]. However, B7-H3 expression is minimal in most normal tissue types but elevated in numerous tumor types (including lung), and as such, it has become an attractive target in ADC design against various malignancies, including SCLC. In addition, within transcriptional subtypes in SCLC, B7-H3 stands out in its ability to be expressed across all subtypes [64].

Trials remain ongoing in studying B7-H3 ADCs in malignancy, with a key ADC being YL201. YL201 comprises a human anti-B7-H3 monoclonal antibody conjugated to a novel topoisomerase I inhibitor payload via a peptide-based, protease-cleavable linker, which undergoes protease-mediated cleavage by lysosomal enzymes following internalization, enabling intracellular payload release. A recent Phase 1/1b trial of YL201 enrolled 312 patients with advanced solid tumors, including 79 patients with ES-SCLC (NCT05434234, NCT06057922). The observed efficacy of YL201 in this ES-SCLC subgroup was particularly remarkable, with an ORR reported at 63.9%, which is notably superior compared to those of standard second-line SCLC therapies [65]. Such therapies include topetecan [66], lurbinectedin [67], and tarlatamab [36], whose ORRs typically range from 21.9% to 40%. The B7-H3 ADC findings are furthered by a mPFS of 6.3 months, as well as a DOR of 5.7 months, which may provide a meaningful clinical benefit within the ES-SCLC population. The most common grade ≥ 3 treatment-related adverse events reported from the YL201 trial included neutropenia (31.7%), leukopenia (29.5%), and anemia (25.0%). Rarer adverse events were also noted, including both interstitial lung disease (1.3%) and infusion reaction (0.3%).

Other challenges, including resistance mechanisms and payload/bystander effect, have yet to be ascertained as trials currently remain ongoing. Such trials include the GSK5764227 trial, which also describes an anti-B7-H3 antibody linked to a topoisomerase inhibitor payload that is currently being evaluated in ES-SCLC and other solid tumors [68]. The GSK5764227 ADC has received the US FDA Breakthrough Therapy Designation for relapsed or refractory ES-SCLC, which has assisted in expediting its development and review [69]. Another recent trial investigates a bispecific ADC (consisting of both DLL3 and B7-H3) that reportedly demonstrated superior in vivo efficacy compared to monovalent ADCs in SCLC cell-derived xenograft models [70]. These ongoing studies will need to be advanced to later-phase trials to better understand the limitations of the B7-H3 ADC in regard to both TME limitations as well as the possibility of combination regimens with immune checkpoint blockade therapy in treating SCLC.

## 6. SEZ6 Antibody–Drug Conjugates

Seizure-related homolog protein 6 (SEZ6) is a type 1 transmembrane protein with biomolecular implications in neuronal development and signaling [71]. As such, its protein expression is primarily restricted to the central nervous system (CNS), with low to negligible physiologic expression in non-neural tissue. In regard to thoracic oncology, studies have also previously demonstrated an overexpression of SEZ6 in SCLC and other high-grade neuroendocrine malignancies compared to that of normal tissues (with the CNS being the key exception) [72]. These characteristics of SEZ6, along with its ability of rapid internalization upon ligand/antibody binding, are favorable attributes in the development of an ADC, thereby prompting further evaluation in the treatment of SCLC. One of the two key trials in the development of SEZ6-targeted ADCs included ABBV-011, a monoclonal antibody (clone SC17) directed to SEZ6, conjugated to calicheamicin (a potent inducer of DNA double-stranded breaks) via a novel-cleavable linker (LD19.10) with a drug-to-antibody ratio (DAR) of 2. LD19.10 is an acid-labile, cleavable linker, designed to remain stable in circulation but undergo intracellular cleavage within lysosomes following SEZ6-mediated internalization, releasing the payload. In vitro studies confirmed that the SC17 antibody binds to SEZ6 prior to internalization and co-localization with lysosomes in engineered SCLC lines (NCT03639194). Furthermore, ABBV-011 demonstrated potent tumor regression in SEZ6-positive models when administered as single doses in mice, while minimal effect was demonstrated in SEZ6-negative models [73].

Following these in vitro and in vivo studies, a multicenter phase I trial of ABBV-011 was conducted in patients (n = 99) with relapsed SCLC (or refractory to 1–3 prior lines), with an ORR of 19%, an mDOR of 4.2 months, and an mPFS of 3.5 months [74]. These findings suggest that although ABBV-011 is a feasible option, response rates remain modest with a short duration of benefit. The most common adverse events included fatigue (50%), nausea (42%), and thrombocytopenia (41%). The trial was also notable for hepatic adverse events, including elevated aspartate aminotransferase (22%), elevated gamma-glutamyltransferase (21%), and hyperbilirubinemia (17%); two patients also experienced veno-occlusive liver disease.

The other key SEZ6-ADC trial involves ABBV-706, a second-generation ADC that targets SEZ6 with a topoisomerase I inhibitor payload conjugated with a cleavable linker and higher DAR of 6 (NCT05599984). The use of different payloads reflects an effort to improve tolerability, as calicheamicin-based ADCs are associated with hepatotoxicity, whereas topoisomerase I inhibitor payloads have shown broader activity with a more manageable safety profile. This trial was a recent phase 1 multicenter study (n = 49), where the ORR was preliminarily found to be 40% within the SCLC subgroup (higher than that of the ABBV-011 trial) [75]. The most common adverse events included anemia (51%), fatigue (41%), and neutropenia (31%).

Limitations in utilizing SEZ6-ADC in treating SCLC have thus far included a modest efficacy of ABBV-011 at best given the low ORR, as well as its associated hepatic toxicity (including the rare but serious risk of veno-occlusive disease) that may associated with the calicheamicin payload. While the remaining results of the ABBV-706 trial are awaiting to be finalized, future studies may require considering new payload choices, further safety monitoring in regard to ABBV-011’s hepatic effects, and combination therapy with either chemotherapy or with immune-checkpoint inhibition.

## 7. Discussion

SCLC remains a disease that has historically been characterized by both rapid relapse as well as poor long-term survival despite various chemotherapy and immunotherapy options. However, the advent of both ADCs and TCEs represents a significant advancement within thoracic oncology based on the premise that all the targeted antigens are highly expressed in malignancies (such as SCLC) but remain minimally expressed in normal tissues. This attribute of tumor-selective overexpression thereby provides an attractive therapeutic window that enables more direct, malignancy-specific targeting compared to standard systemic treatments such as chemotherapy. DLL3, namely, remains the most extensively studied among these antigens, with TCEs such as tarlatamab producing effective and potent immune activation. In early clinical trials, tarlatamab has demonstrated meaningful ORRs as well as a manageable safety profile (i.e., low-grade CRS). These findings resulted in its FDA traditional approval that ultimately established DLL3 as a clinically validated target in treating SCLC.

DLL3-targeted ADCs, however, have generated mixed results thus far, as the efficacy results of Rova-T were limited by an unfavorable toxicity profile, while ZL-1310 demonstrated both effective activity as well as a more manageable safety profile. This contrast underscores the importance of payload selection, linker stability, DAR, and antigen density in engineering the ADC. Early clinical trials have also established encouraging activity in the newer ADC platforms (targeting TROP2, B7-H3, and SEZ6). TROP2-ADCs, hallmarked by SG and DXd constructs (both containing potent topoisomerase I payloads), demonstrated significant tumor response across both platinum-sensitive and platinum-resistant SCLC. Similarly, YL201, the hallmark B7-H3 ADC, has yielded compelling response rates in extensive-stage disease. SEZ6 ADCs provide additional promise, with ABBV-706 offering more favorable efficacy results compared to its earlier-generation ABBV-011 counterpart. This myriad of ADC engineering strategies collectively illustrate how diverse antigen targets may further enlarge the therapeutic toolkit in treating SCLC.

In parallel with antibody-based approaches, chimeric antigen receptor T-cell (CAR-T) therapies represent a personalized cellular immunotherapy strategy involving ex vivo genetic modification of autologous T cells to target tumor-associated antigens. In SCLC, early investigational efforts are exploring CAR-T constructs directed against targets such as CDH17, GD2, and PTK7, which are selected based on tumor-enriched expression patterns similar to those leveraged by ADCs and T-cell engagers [76,77,78]. Recently, LB2102, a DLL3-directed CAR-T therapy engineered with a TGF-β receptor blockade, has shown an acceptable early safety profile and preliminary dose-dependent antitumor activity in relapsed and refractory SCLC, with evidence of CAR-T expansion and a partial response observed at higher dose levels [79]. Although CAR T therapies pose distinct challenges related to manufacturing complexity, toxicity, and tumor microenvironment barriers, they may complement antibody-based immunotherapies through shared antigen targets and potential combinatorial strategies.

Emerging ADC and T-cell-based targets in SCLC align closely with the disease’s underlying lineage and molecular heterogeneity. Neuroendocrine lineage-associated antigens such as DLL3 are highly expressed in SCLC and closely linked to ASCL1- and NEUROD1-driven transcriptional programs, which define the predominant neuroendocrine subtypes (SCLC-A and SCLC-N) [6,80,81]. In contrast, SCLC exhibits marked plasticity driven by oncogenic factors such as MYC and frequent alterations in epigenetic regulators including CREBBP, EP300, and KMT2 family genes, which have been shown to influence lineage state transitions and therapeutic resistance [82,83]. These features underscore the importance of incorporating molecular subtype and lineage context when developing and evaluating targeted immunotherapies in SCLC, particularly given the coexistence of non-neuroendocrine phenotypes such as SCLC-P and SCLC-I.

## 8. Limitations and Future Directions

Several limitations must be addressed as clinical trials continue to make progress with these various ADC strategies. One such limitation is the prevalence of toxicity, as reflected by the cytokine-release syndrome and neurotoxicity observed with TCEs as well as by the hematologic and hepatic adverse events observed with some second-generation ADCs. Furthermore, durability of response may be limited by intratumoral heterogeneity and dynamic antigen expression. Finally, the early-phase trials have not thoroughly evaluated mechanisms of ADC resistance, and as such, the possibility of antigen downregulation and alterations in the tumor microenvironment remain poorly understood. More robust biomarker development to both identify the correct therapy for the patient’s SCLC tumor and to better acquired resistance from these agents is therefore recommended to better delineate which patients are most likely to benefit from ADC-targeted therapies. Furthermore, this development will likely require strategies beyond the current biomarker strategy of using next-generation sequencing and IHC H-scores. This is critical as there are a rapidly growing number of new therapies and gene targets yet given the drug and financial toxicities of these agents, it is important that we prudently select these agents which can happen with reliable biomarkers.

As clinical trials remain ongoing to further assess the various ADC platforms, other next steps in the development of these agents will likely focus on optimizing patient selection as well as refinement in the payload and linker chemistry in order to mitigate excess toxicity. Furthermore, incorporation of biomarker stratification within clinical trial designs is recommended to help better capture SCLC’s immunologic diversity and evaluate for possible mechanisms of ADC resistance. In addition, with multiple agents and gene targets, sequencing of these drugs or exploring even combinations of the drugs together based on acquired resistance patterns could play a pivotal role. Finally, given the innate immunologic effects of both ADCs and TCEs, combining them with other agents—such as immune checkpoint inhibitors, PARP inhibitors, and systemic chemotherapy—should be further investigated in future clinical trials. These combination strategies should ideally be compared against different standard monotherapy strategies in terms of both efficacy and toxicity.

## 9. Conclusions

ADCs and TCEs are beginning to reshape the general approach in treating SCLC by enabling precise, antigen-specific targeting against a disease that notably remains difficult to treat. Although important challenges remain in refining tumor response rates and mitigating toxicity rates, both the early success of TCEs as well as the growing panel of numerous promising ADC platforms reflect a meaningful shift in the SCLC therapeutic landscape. Continued innovation in antigen targeting, payload/linker chemistry, and combination-therapy trials will be paramount in order to both advance these novel immunotherapies and ultimately improve outcomes in SCLC patients.

## Figures and Tables

**Table 1:** List of Clinical Trial Results for Antibody–Drug Conjugates and T-Cell Engagers.

Target	Drug	Trial (Number, Phase)	Target Population	Results
DLL3	Tarlatamab (AMG 757)	DeLLphi-300 (NCT03319940, I)	Relapsed SCLC; dose exploration (n = 73) and expansion (100 mg, n = 34)	ORR 23.4% mDOR 12.3 mo mPFS 3.7 mo mOS 13.2 mo
		DeLLphi-303 (NCT05361395, I)	Tarlatamab and atezolizumab (n = 48) vs. tarlatamab and durvalamab (n = 40) as first-line maintenance in ES-SCLC	mPFS 5.6 mo DCR 62.5%
		NCT04885998, Ib	Tarlatamab + AMG 404 (n = 14)	ORR 20.0–66.7%
		DeLLphi-301 (NCT05060016, II)	SCLC with median 2 previous lines of therapy (n = 110 on 10 mg, n = 110 on 100 mg)	10 mg vs. 100 mg: ORR 55% vs. 57% mPFS 4.9 vs. 3.9 mo
		DeLLphi-304 (NCT05740566, III)	Tarlatamab (n = 254) vs. SOC (n = 255) in progressed/relapsed SCLC	ORR 35% vs. 20% mOS 13.6 mo vs. 8.3 mo mPFS 4.2 mo vs. 3.7 mo
		DeLLphi-305 (NCT06211036, III)	Tarlatamab and durvalamab vs. durvalumab as maintenance after 1 L in ES-SCLC (n = 563)	Not reported
		DeLLphi-306 (NCT06117774, III)	Tarlatamab vs. placebo in LS-SCLC following chemoradiation (n = 400)	Not reported
		DeLLphi-312 (NCT07005128, III)	Tarlatamab + SOC vs. SOC (durvalumab, carboplatin, etoposide) in untreated ES-SCLC (n = 330)	Not reported
	Obrixtamig (BI764532)	NCT04429087, I	DLL3 positive ES-SCLC and neuroendocrine carcinoma (n = 300)	SCLC (n = 24): ORR 33%
		DAREON-7 (NCT06132113, I)	DLL3 positive neuroendocrine cancer (n = 55)	Not reported
		DAREON-8 (NCT06077500, I)	BI 764532 + SOC in ES-SCLC (n = 46)	Not reported
		DAREON-9 (NCT05990738, Ib)	BI 764532 + topotecan in relapsed ES-SCLC (n = 25)	Unconfirmed ORR 70% DCR 87%
		NCT05879978, I	BI 764532 + ezabenlimab in DLL3-positive progressive ES-SCLC (n = 45)	Not reported
	QLS31904	NCT05461287, I	QLS31904 in advanced solid tumors (n = 290, SCLC-specific unknown)	Not reported
	MK-6070 (HPN 328, gocatamig)	NCT04471727, I/II	MK-6070 monotherapy, MK-6070 + atezolizumab, MK-6070 + I-DXd in DLL3-positive advanced cancers (n = 232, SCLC-specific unknown)	Not reported
		NCT06780137, I/II	Gocatamig monotherapy, gocatamig + I-DXd, gocatamig + durvalumab in unresponsive SCLC (n = 242)	Not reported
	RO7616789	NCT05619744, I	Relapsed ES-SCLC and Advanced neuroendocrine carcinoma (n = 41)	Not reported
	ZG006	NCT05978284, I/II	Unresponsive ES-SCLC and Advanced neuroendocrine carcinoma (n = 54)	ES-SCLC (n = 23): ORR 60.9% DCR 78.3%
	Rova-T	NCT01901653, I	Previously treated ES-SCLC and neuroendocrine tumor (n = 82)	ORR 18% (High DLL3: 38%)
		TRINITY (NCT02674568, II)	Rova-T as third-line and beyond for ES-SCLC	ORR 12.4% mOS 5.6 mo
		TAHOE (NCT03061812, III)	Rova-T (n = 296) vs. topotecan (n = 148) as second-line for DLL3-high ES-SCLC	mOS 6.3 vs. 8.6 mo
	ZL-1310	NCT06179069, I	ZL-1310 monotherapy, ZL-1310 + atezolizumab +/− carboplatin (n = 112) in progressed ES-SCLC	ZL-1310 (n = 28): ORR 68%
	IBI3009	NCT06613009, I	Unresectable, Metastatic, or ES-SCLC	Not reported
	SHR-4849	NCT06443489, I	Advanced solid tumor	Not reported
	FZ-AD005	NCT06424665, I	Advanced solid tumor	Not reported
TROP2	Sacituzumab Govitecan (SG)	TROPiCS-03 (NCT03964727, II)	ES-SCLC with 1 previous line of platinum-based therapy and PD-L1 therapy (10 mg/kg, n = 43)	ORR 41.9% mDOR 4.73 mo mPFS 4.40 mo mOS 13.6 mo
	Ifinatamab deruxtecan (I-DXd)	IDeate-Lung01 (NCT05280470, II)	ES-SCLC with 1 previous line of platinum-based therapy and ≤3 previous lines of systemic therapy (12 mg/kg, n = 137)	ORR 48.2% mDOR 5.3 mo mPFS 4.9 mo 9-mo OS rate 59.1%
B7-H3	YL201	NCT05434234, I NCT06057922, I	ES-SCLC with median 1 previous line of therapy; dose-escalation and expansion (n = 72)	ORR 63.9% mPFS = 6.3 mo mDOR 5.7 mo
SEZ6	ABBV-011	NCT03639194, I	SCLC with ≤3 lines of prior therapy, including ≥1 prior platinum-containing chemotherapy (n = 99)	ORR 19% mDOR 4.2 mo mPFS 3.5 mo
	ABBV-706	NCT05599984, I	SCLC with ≥1 prior platinum-containing chemotherapy (n = 22)	ORR 73% (confirmed and unconfirmed)

ORR: overall response rate; mDOR: median duration of response; mPFS: median progression-free survival; mOS: median overall survival; ES-SCLC: extensive stage-small-cell lung cancer; SOC: standard of care.

## Data Availability

No new data were created or analyzed in this study.

## Supplementary Materials

The following supporting information can be downloaded at: https://www.mdpi.com/article/10.3390/antib15010004/s1, Figure S1: Structure and Mechanism of Action of T-Cell Engagers and Antibody Drug Conjugates.

## Abbreviations

The following abbreviations are used in this manuscript:

SCLC	Small-cell lung cancer
NSCLC	Non-small-cell lung cancer
LS-SCLC	Limited-stage small-cell lung cancer
ES-SCLC	Extensive-stage small-cell lung cancer
ADC	Antibody drug conjugate
TCE	T-Cell engager
ORR	Overall response rate
mDOR	Median duration of response
mPFS	Median progression-free survival
mOS	Medial overall survival
SOC	Standard of care
DLL3	Delta-like ligand 3
TROP2	Trophoblast cell surface antigen-2
SG	Sacituzumab Govitecan
I-DXd	Ifinatamab deruxtecan
TME	Tumor microenvironment
SEZ6	Seizure-related homolog protein 6
