# Supplementary figures and images for: Updates on Antibody Drug Conjugates and Bispecific T-Cell Engagers in SCLC
Source: Antibodies (Basel). 2026 Jan 4;15(1):4. doi: 10.3390/antib15010004 (PMC12821401; doi:10.3390/antib15010004)

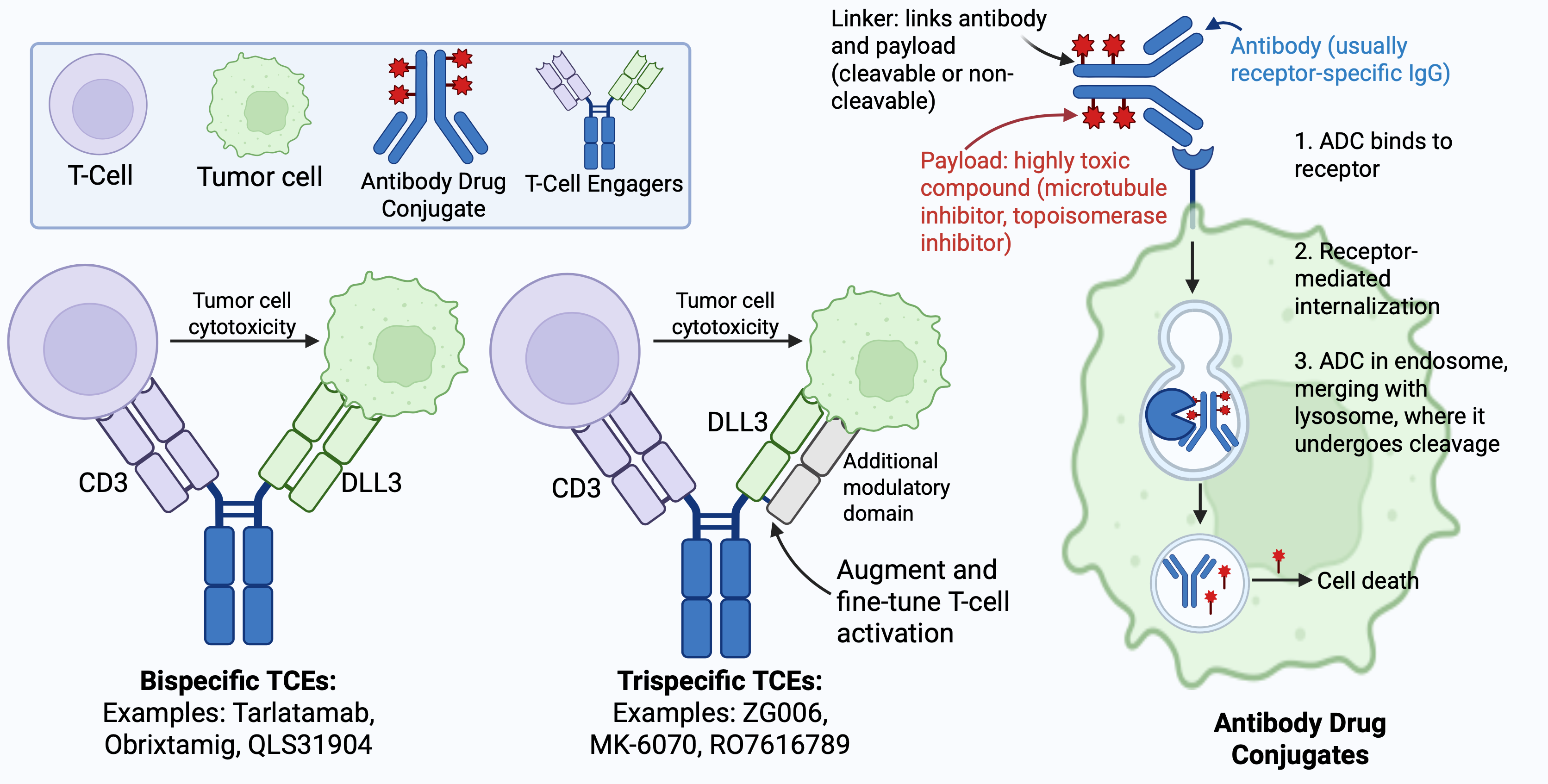

Supplement: Supplementary file 1 [file antibodies-15-00004-s001.zip › antibodies-4049311-supplementary/antibodies-4049311-supplementary Figure S1.png]
